# The impact of statin therapy on the prognosis of hormone receptor-positive breast cancer: a systematic review and meta-analysis

**DOI:** 10.3389/fonc.2026.1829654

**Published:** 2026-06-03

**Authors:** Xiaowen Ma, Yiming Sun, Yaqin Qi, Hiajiang Qu, Feng Zhang

**Affiliations:** 1 Department of Thyroid and Breast Surgery, Hangzhou Women’s Hospital, Hangzhou, Zhejiang, China; 2 Department of Neurosurgery, Tongde Hospital of Zhejiang Province, Hangzhou, Zhejiang, China

**Keywords:** breast cancer, hormone receptor-positive, meta-analysis, prognosis, statins, tumor recurrence

## Abstract

**Background:**

Statins are increasingly recognized for their potential to reduce endocrine therapy resistance and improve overall survival in breast cancer. However, their specific prognostic impact on hormone receptor-positive (HR+) breast cancer remains incompletely understood. This study aims to evaluate the effect of statin use on recurrence and mortality in HR+ breast cancer patients through a comprehensive meta-analysis.

**Methods:**

We systematically searched PubMed, Medline, Cochrane Library, Embase, and Web of Science for studies comparing the outcomes of statin users versus non-users among HR+ breast cancer patients. Hazard ratio (HR) and 95% confidence intervals (CIs) were pooled using appropriate statistical models.

**Results:**

The meta-analysis incorporated 7 studies encompassing 267,913 HR+ breast cancer patients. Pooled analyses demonstrated that statin use was significantly associated with a 23% reduction in recurrence risk (HR = 0.77; 95% CI: 0.61–0.98, P = 0.03) and a significant decrease in mortality (HR = 0.77; 95% CI: 0.73–0.81, P < 0.001). Sensitivity analyses confirmed the robustness of these findings.

**Conclusion:**

Our large-scale meta-analysis suggests that statin therapy confers a protective effect against recurrence and mortality in HR+ breast cancer. These findings highlight the promising role of statins as an effective adjunctive therapy and underscore the necessity for future large-scale, prospective trials to optimize their clinical applications.

**Systematic review registration:**

https://www.crd.york.ac.uk/PROSPERO/view/CRD42024599286, identifier CRD42024599286.

## 1 Introduction

Statins (HMG-CoA reductase inhibitors) are foundational for cardiovascular disease (CVD) prevention and management, including primary prevention in high-risk patients (e.g., diabetes, familial hypercholesterolemia), secondary prevention of recurrent events (e.g., myocardial infarction, stroke), and first-line treatment of hypercholesterolemia. They mitigate atherosclerotic CVD (ASCVD) progression through plaque stabilization and improve outcomes in acute coronary syndrome (ACS), peripheral artery disease (PAD), and ischemic stroke prevention (1). Beyond their potent lipid-lowering capabilities, statins exert significant pleiotropic effects, such as reducing inflammation (lowered CRP), improving endothelial function, and potentially reducing the risk of venous thrombosis (2).

Emerging evidence suggests that these pleiotropic properties may translate into anti-tumor potential in breast cancer, improving survival outcomes across various subtypes, including hormone receptor-positive (HR+) and triple-negative cancers (3). However, the rationale for investigating statin efficacy is particularly compelling for HR+ breast cancer, which accounts for approximately 70-80% of all breast cancer diagnoses. While adjuvant endocrine therapy has drastically improved the prognosis for HR+ patients, acquired endocrine resistance remains a leading cause of tumor recurrence and mortality.

Recent research highlights a critical intersection between cholesterol metabolism and estrogen receptor signaling. Since cholesterol serves as an essential precursor for estrogen synthesis, and the overactivation of the mevalonate pathway is heavily implicated in tumor proliferation, statins are hypothesized to uniquely benefit HR+ patients. By inhibiting HMG-CoA reductase, statins can deprive tumors of crucial lipid precursors and actively mitigate endocrine therapy resistance (4).

Despite this strong biological plausibility, clinical and epidemiological evidence regarding the prognostic impact of statins specifically in HR+ breast cancer remains inconsistent and limited across different clinical stages. Therefore, this study conducts a comprehensive meta-analysis to systematically evaluate the impact of statin therapy on recurrence and mortality specifically in HR+ breast cancer patients, aiming to clarify its potential as an adjunctive anti-tumor strategy.

## 2 Methods

### 2.1 Study design and guidelines

This study was a systematic review and meta-analysis. The research was conducted in accordance with the PRISMA 2020 statement and MOOSE recommendations for observational studies. The prespecified outcomes were recurrence and survival-related outcomes, including overall survival (OS), breast cancer-specific survival (BCSS), and mortality as reported by the original studies. Importantly, recurrence analyses were restricted to non-metastatic populations, whereas studies including stage IV disease were considered only for survival-related analyses.

### 2.2 Literature search strategy

A comprehensive literature search was independently conducted by three reviewers (Xiaowen Ma, Yiming Sun, and Yaqin Qi) across multiple databases, including PubMed, Medline, Embase, Cochrane Library, and Web of Science. The search spanned from January 1, 2005, to January 1, 2026. The search employed established medical subject headings (MeSH) and keywords: [“statin”(All Fields) OR “Hydroxymethylglutaryl-CoA Reductase Inhibitors”(MeSH Terms)] AND [“breast neoplasms”(MeSH Terms) OR “breast cancer”]. All identified citations were independently assessed, and reference lists of selected full-text articles were manually screened for additional relevant studies. Ecological studies, case reports, reviews, and editorials were excluded.

### 2.3 Eligibility criteria

Studies were included if they met the following criteria:(1) they reported adjusted hazard ratios (HRs) with corresponding 95% confidence intervals (CIs) for recurrence and/or survival outcomes comparing statin users with non-users; (2) they included patients with hormone receptor-positive (HR+) breast cancer; and (3) they were original, non-overlapping cohort studies. For the recurrence analysis, only studies evaluating non-metastatic disease (generally stage I–III) were considered eligible. Studies including stage IV disease were retained only for survival-related analyses, provided that the reported endpoint was mortality, overall survival (OS), or breast cancer-specific survival (BCSS). “Recurrence” was defined as local, regional, distant, or contralateral breast cancer events, according to the original study definitions. Statin exposure was defined as post-diagnostic statin use, irrespective of specific agent, dose, or lipophilicity/hydrophilicity.

### 2.4 Data extraction

Three reviewers (Xiaowen Ma, Yiming Sun, and Yaqin Qi) independently extracted data using a standardized collection protocol, resolving any discrepancies through consensus. Extracted data included lead author, publication year, study design, geographical context, median follow-up period, patient characteristics (median age, tumor stage, nodal stage), definitions of statin use, and adjusted HRs for recurrence or death. Cancer stage was documented based on the applicable American Joint Committee on Cancer (AJCC) manual at the time of diagnosis.

### 2.5 Eligibility for synthesis

Reviewers decided study eligibility for each synthesis by tabulating characteristics and comparing them against the prespecified analytic groups. Recurrence analyses were strictly restricted to non-metastatic (Stage I–III) populations. Studies that included Stage IV disease were excluded from recurrence syntheses but retained for survival-related syntheses (OS, BCSS, or mortality). Any studies using endocrine therapy only as a proxy for hormone receptor status without explicit HR+ confirmation were excluded.

### 2.6 Data preparation

To prepare the data for synthesis, fully adjusted HRs and their 95% Confidence Intervals (CIs) were directly extracted from the original texts. Data conversions were performed by calculating the natural logarithms of the HRs and their corresponding standard errors. Odds Ratios (ORs) were excluded as they are considered inappropriate for time-to-event survival data.

### 2.7 Tabulation and visual display

Study results and syntheses were visually displayed through the following methods:

Tables: Key characteristics of the included studies, recurrence/survival statistical results, and quality assessment (NOS) scores were tabulated.

Forest Plots: Used to illustrate pooled effects for recurrence, mortality, and survival subgroups.

Funnel Plots: Employed to visually assess publication bias.

Kaplan–Meier Curves: Utilized to display recurrence-free survival in the real-world cohort.

Synthesis Methods and Rationale: Statistical synthesis was performed using a random-effects model (DerSimonian-Laird method), chosen to account for potential variation across diverse global cohorts. Statistical heterogeneity was identified using the Cochran Q test and quantified with the I^2^ statistic, where I^2^ < 50% was defined as acceptable variability. All meta-analyses were conducted using RevMan version 5.4 and Stata version 17.

Exploration of Heterogeneity: Causes of heterogeneity were explored through subgroup analyses based on: Clinical Stage: Comparing early-stage (I–III) versus mixed-stage (I–IV) cohorts. Specific Endpoints: Differentiating between all-cause mortality and breast cancer-specific survival (BCSS). Qualitative exploration also considered differences in age distribution, geographical context, and statin lipophilicity.

### 2.8 Sensitivity analysis

To assess the robustness of the findings, leave-one-out sensitivity analyses were conducted. This involved sequentially omitting each individual study and recalculating the pooled HR to ensure that no single study disproportionately influenced the overall conclusions regarding recurrence or mortality.

#### 2.8.1 Certainty assessment

Methods for assessing the certainty of the body of evidence included evaluating the methodological quality of the studies, analyzing result consistency, and performing robustness tests. We utilized the Newcastle-Ottawa Scale (NOS) to assess the quality of the seven included cohort studies across three domains: selection, comparability, and outcome assessment. All included studies achieved high-quality scores of 7 to 8 points, reflecting a low risk of bias and robust methodological rigor. The certainty of the evidence was further evaluated by examining the consistency of outcomes using the I^2^ statistic and Cochran Q test, which revealed exceptional consistency for mortality data (I^2^ = 0%). To ensure the stability of the synthesized results, leave-one-out sensitivity analyses were conducted for both recurrence and mortality outcomes, confirming that the overall estimates were not disproportionately influenced by any single study. Additionally, publication bias was assessed through funnel plots and Egger’s regression test.

For all analyses, statistical significance was defined as a two-sided *P* < 0.05, or when the 95% CI excluded 1.00.

## 3 Result

### 3.1 Results of the search strategy

**Figure 1:**
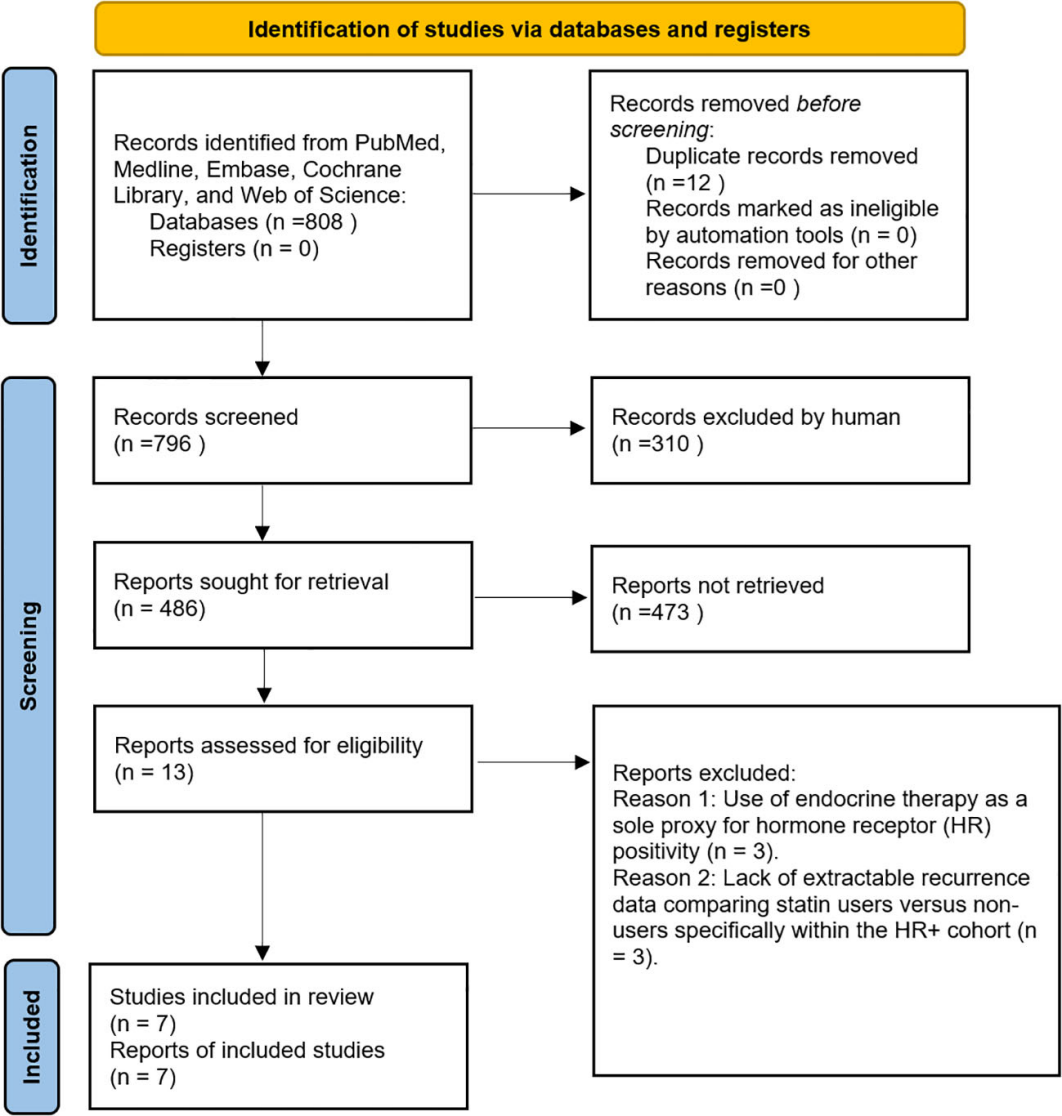
Flowchart of the article selection process.

A total of 796 articles were identified using the specified search strategy and MeSH terms. Following the screening of titles and abstracts, 310 articles were excluded, leaving 486 articles for full-text review. Upon detailed evaluation of 13 potentially eligible studies, 6 were further excluded: 3 lacked extractable recurrence data comparing statin users and non-users specifically within the HR+ patient cohort, and 3 were excluded due to the use of endocrine therapy merely as a proxy for hormone receptor positivity. Ultimately, 7 studies met all inclusion criteria and were incorporated into the meta-analysis (**Figure 1**).

### 3.2 Description of studies

**Table 1** summarizes the key characteristics of the seven included studies, including country, study design, stage distribution, follow-up duration, and reported endpoints. This table presents a thorough summary, including the total number of cases, publication years, geographical regions, diagnostic periods, study designs, clinical stages, median follow-up durations, and endpoints. A total of 7 cohort studies involving 267,913 patients with HR+ breast cancer were included. The included studies were published between 2011 and 2025 and represented populations from New Zealand, Singapore, Scotland, Denmark, Sweden, the United States, and Taiwan. Most studies were retrospective cohorts, with one prospective cohort. Median follow-up ranged from 3.7 to 8.7 years. Four studies contributed recurrence data, all of which were restricted to stage I–III or otherwise non-metastatic HR+ populations. Five studies contributed survival-related outcomes, including BCSS or OS; among these, two studies included stage I–IV patients and were retained only for the survival analysis. This stage-specific analytic strategy was applied to avoid mixing metastatic disease into the recurrence endpoint.

**Table 1:** Characteristics of the seven included studies of HR+ breast cancer.

First author	Public year	Country and diagnostic time	Study type	Cases	Controls	BC stage	Median follow-up(months/years)	Endpoints
Scott OW (5)	2023	New Zealand 2007-2016	retrospective cohort	12264	NA	I-IIIa	4.5y	BCSS
Sim Y (6)	2022	Singapore 2005-2015	retrospective cohort	5000	NA	I-III	8.7y	BCSS and recurrence
Mc Menamin ÚC (7)	2016	Scottish 2009 to 2012	retrospective cohort	12650	NA	I-IV	4y	BCSS
Ahern TP (8)	2011	Denmark 1996-2003	prospective cohort	14200	NA	I-III	6.8y	Recurrence
Borgquist S (9)	2017	Sweden 1998-2003	retrospective cohort	7963	postmenopausal	I-III	8y	Recurrence
Hanbing Guo (3)	2024	USA2008-2017	retrospective cohort	30022	>65y, HER2-	I-III	3.7y	BCSS and recurrence
Chen C-F (10)	2025	TriNetX 2010-2020	retrospective cohort	185814	NA	I-IV	5y	OS

**Table 2** provides a comparative analysis of total patient numbers, recurrence incidences and rates, adjusted HRs, and P-values between stage I-III HR+ breast cancer patients using statins versus those not using statins across four included studies. In three out of the four studies (Ahern TP, Sim Y, and Borgquist S), the adjusted HRs were significantly below 1.00, with 95% confidence intervals excluding 1.00 and P-values < 0.05. These findings indicate a significantly lower risk of recurrence among HR+ patients on statin therapy. However, one study (Hanbing Guo) reported a non-significant adjusted HR of 1.03 (95% CI: 0.83–1.28), demonstrating no clear recurrence benefit in that specific cohort. Despite this minor variability, the majority of the extracted data consistently demonstrates lower recurrence rates among HR+ breast cancer patients receiving statin therapy, strongly suggesting the potential protective benefits of statin use in this patient population.

**Table 2:** The recurrence values and statistical results of each study.

First author	Statins use			No use			Adjusted HR and CI	P
	Total case (n)	Recurrence case	Recurrence rate (%)	Total case (n)	Recurrence case	Recurrence rate (%)		
Ahern TP (8)	2613	198	7.58	11587	2174	18.75	0.69 (0.55-0.88)	<0.05
Sim Y (6)	851	59	6.93	4149	773	18.63	0.57 (0.43-0.76)	<0.001
Borgquist S (9)	637	124	19.47	7326	1881	25.68	0.84(0.68-0.99)	0.04
Hanbing Guo (3)	3693	NA	NA	14349	NA	NA	1.03(0.83-1.28)	NA

**Table 3** provides a comparative analysis of total patient numbers, breast cancer deaths, mortality rates, adjusted HRs, and P-values between stage I-IV HR+ breast cancer patients using statins versus those not using statins across five included studies. In four out of the five studies (Scott OW, Sim Y, Hanbing Guo, and Chen C-F), the adjusted HRs were significantly below 1.00, with 95% confidence intervals excluding 1.00. These findings consistently indicate significantly lower mortality rates and improved survival outcomes for HR+ patients receiving statin therapy. However, the study by Mc Menamin ÚC reported an adjusted HR of 0.92 (95% CI: 0.72–1.16) with a P-value of 0.48, showing no statistically significant difference in mortality risk based on statin use within their specific cohort. Despite this single non-significant finding, the overwhelming majority of the data strongly supports a substantial survival benefit and reduced mortality risk associated with statin therapy in HR+ breast cancer patients.

**Table 3:** The survival values and statistical results of each study.

First author	Statins use			No use			Adjusted HR and CI	P
	Total case (n)	Breast cancer deaths	Death rate (%)	Total case (n)	Breast cancer deaths	Death rate (%)		
Scott OW (5)	3361	165	4.91	8933	654	7.32	0.74(0.63–0.88)	<0.05
Sim Y (6)	851	56	6.58	4149	415	10.00	0.72(0.53 – 0.97)	0.028
Mc Menamin ÚC (7)	3655	243	6.65	8995	552	6.14	0.92(0.72,1.16)	0.48
Hanbing Guo (3)	5118	NA	NA	19020	NA	NA	0.71(0.57-0.88)	NA
Chen C-F (10)	93236	10412	11.17	92578	14332	15.48	0.766(0.723-0.811)	<0.001

**Table 4** details the quality assessment of the seven included studies using the Newcastle-Ottawa Scale (NOS). All studies achieved high-quality scores ranging from 7 to 8 points (out of a maximum of 9), reflecting robust methodological rigor. Notably, these studies demonstrated excellent performance across the core domains of cohort selection, comparability, and outcome assessment. By effectively adjusting for key confounding variables and minimizing potential selection biases, the included cohorts yield highly reliable and credible evidence for this meta-analysis.

**Table 4:** Newcastle–Ottawa Scale (NOS).

Study	Selection				Comparability	Outcome			
	Representativeness of the exposed cohort	Selection of the nonexposed cohort	Ascertainment of exposure	Demonstration that outcome of interest was not present at start of study	Comparability of cohorts on the basis of the design or analysis	Assessment of outcome	Was follow-up long enough for outcomes to occur	Adequacy of follow-up of cohorts	Quality score
Ahern TP (8)	★		★	★	★★	★	★	★	8
Sim Y (6)	★		★	★	★★	★	★	★	8
Borgquist S (9)	★		★	★	★★	★	★	★	8
Scott OW (5)	★		★	★	★★	★	★		7
Mc Menamin ÚC (7)	★		★	★	★★	★	★		7
Hanbing Guo (3)	★		★	★	★★	★	★	★	8
Chen C-F (10)	★		★	★	★★	★	★		7

### 3.3 Meta-analysis Results

#### 3.3.1 Impact of statin use on recurrence risk in HR+ breast cancer

**Figure 2:**
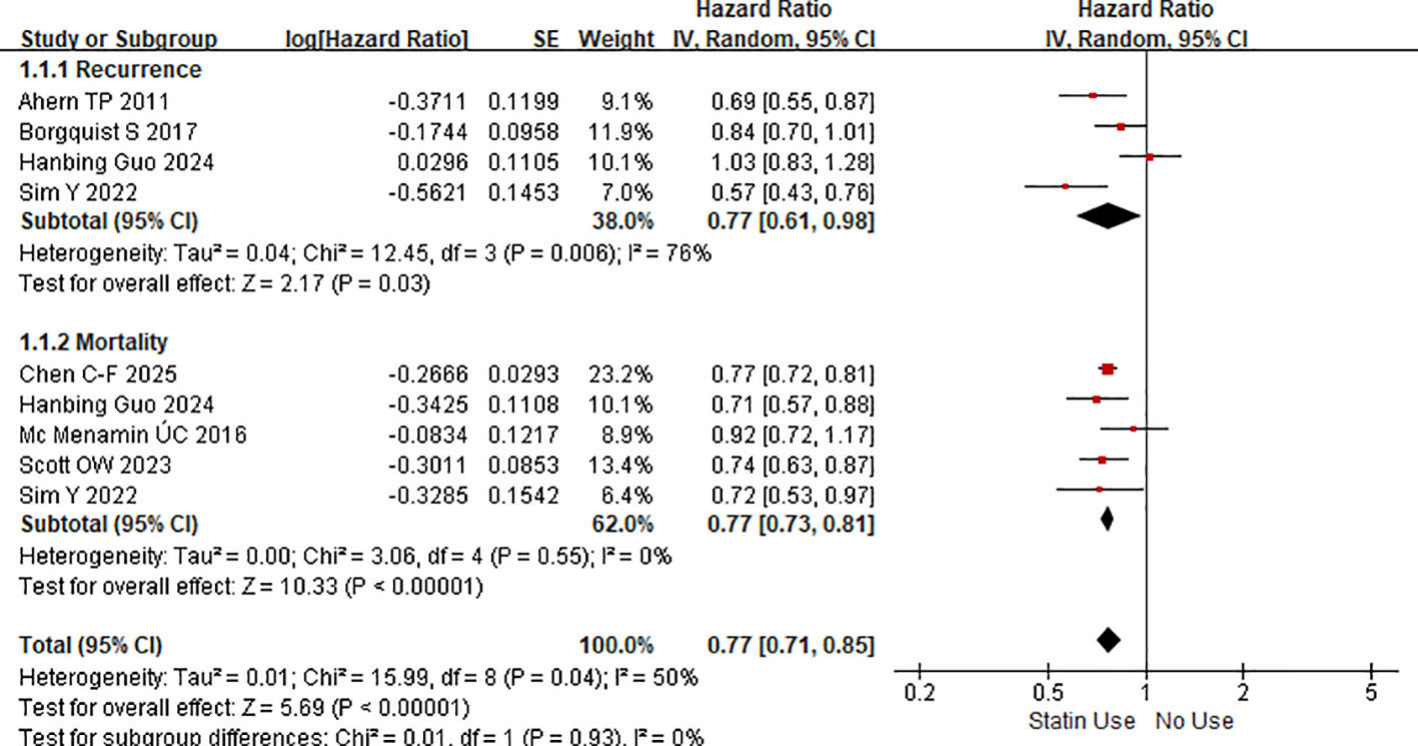
The forest plot compares the effects of statin use versus no use on the risk of recurrence and mortality in hormone receptor-positive breast cancer.

The pooled analysis reveals a notably significant 23% reduction in recurrence risk for patients on statin therapy compared to those not on statins (HR = 0.77; 95% CI: 0.61–0.98, P = 0.03), as illustrated in **Figure 2**. This finding strongly suggests that stage I-III HR+ breast cancer patients who receive statins exhibit a substantial decrease in recurrence rates. However, it is crucial to acknowledge the substantial heterogeneity observed among these included studies, with an I^2^ statistic of 76% and a Chi-square test P = 0.006.

#### 3.3.2 Impact of statin use on mortality in HR+ breast cancer

As illustrated in **Figure 2**, the pooled HR for mortality is 0.77 (95% CI: 0.73–0.81), indicated by the diamond which lies completely to the left of the line of no effect (HR = 1). The overall effect test (Z = 9.87, P < 0.00001) confirms that statin use has a highly statistically significant impact on improving OS and BCSS in stage I-IV HR+ breast cancer patients, reducing the risk of mortality by 23%.

Heterogeneity Assessment: In stark contrast to the recurrence analysis, the mortality analysis demonstrated minimal to no heterogeneity among the five included studies (Tau^2^ = 0.00, Chi^2^ = 3.39, df = 4, I^2^ = 0%, P = 0.50). An I^2^ of 0% highlights exceptional consistency across the diverse cohorts, indicating that the observed survival benefit of statins is highly robust and not influenced by variations in study characteristics.

#### 3.3.3 Publication bias

**Figure 3:**
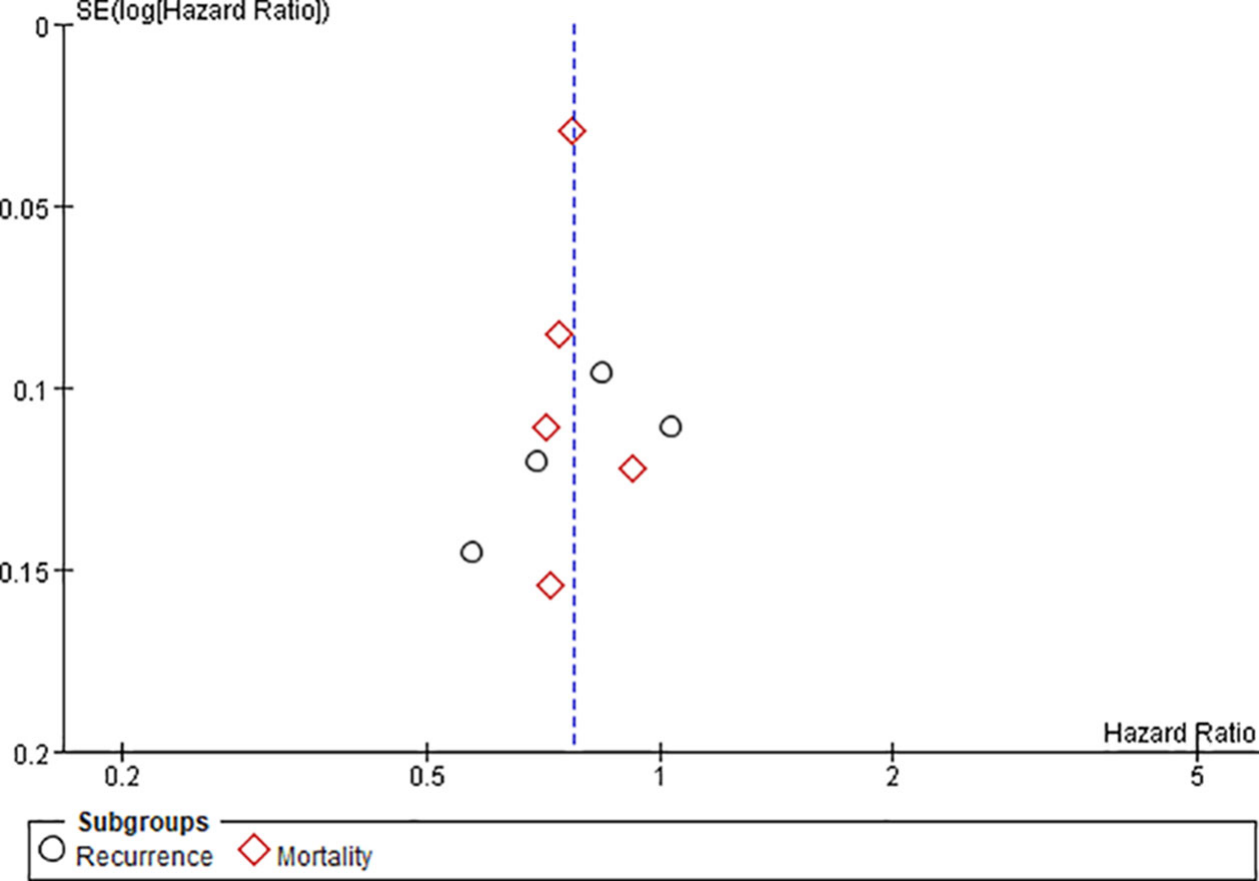
Funnel plot for assessment of publication bias in studies reporting hazard ratios for recurrence and mortality.

Publication bias was assessed using funnel plot visualization and Egger’s regression test for both recurrence and mortality outcomes. For recurrence, the funnel plot (**Figure 3**) showed that studies (circles) were distributed approximately symmetrically around the pooled effect estimate and within the expected funnel region, suggesting no substantial publication bias. Egger’s regression test for recurrence confirmed no significant small-study effects (P = 0.39).

**Figure 4:**
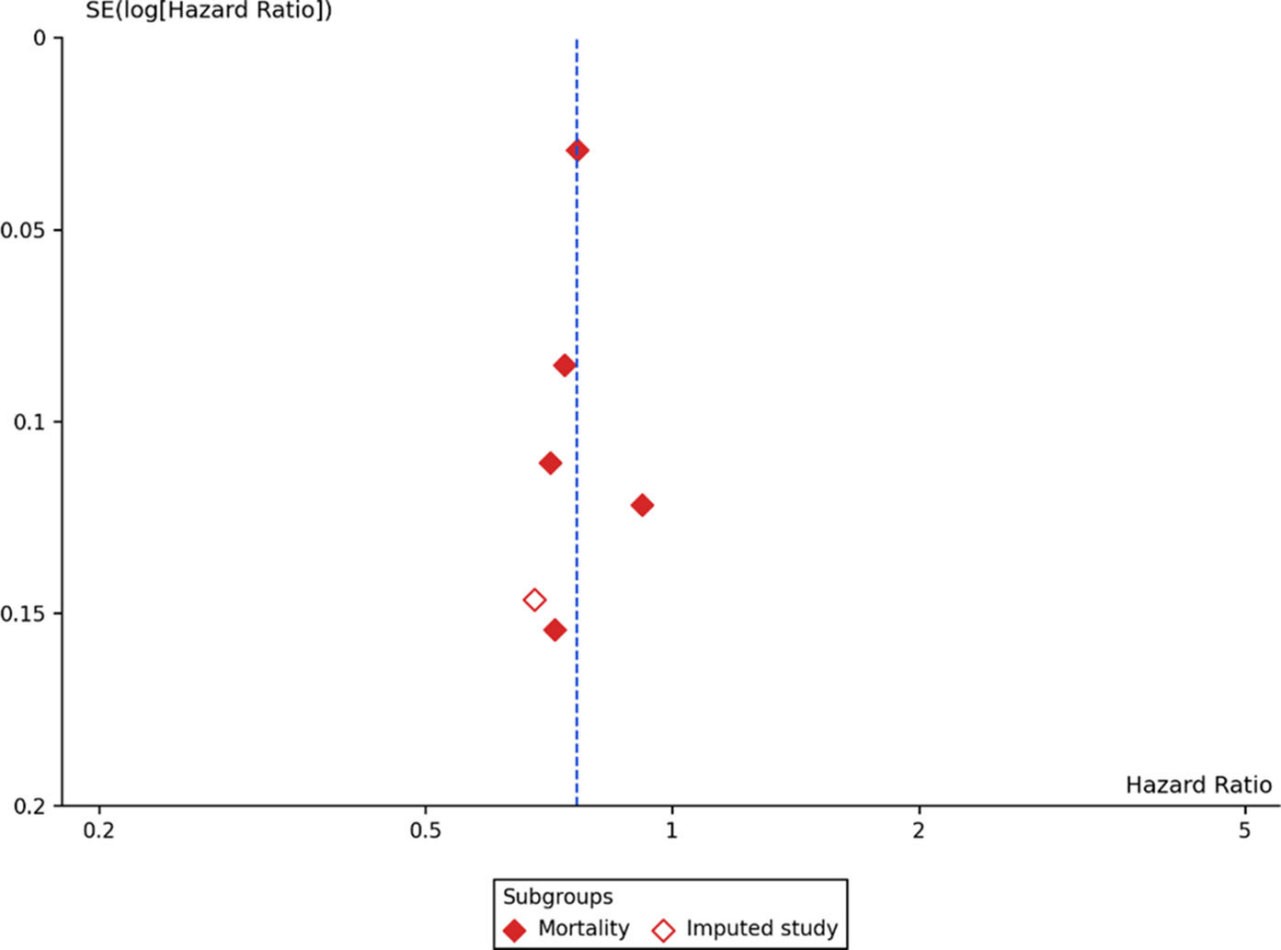
Funnel plot for mortality outcomes with trim and fill adjustment.

For mortality outcomes, the funnel plot (**Figure 4**) did not show visually obvious asymmetry, but Egger’s regression test was statistically significant (P = 0.009), indicating potential small-study effects. To evaluate the impact of possible missing studies, a Trim and Fill analysis was performed, which imputed one hypothetical study. The adjusted pooled hazard ratio changed minimally to HR = 0.765 (95% CI: 0.728–0.805), indicating that the protective effect of statin use on mortality remained robust even after accounting for potential publication bias.

It should be noted that the number of included studies for both recurrence and mortality analyses was fewer than ten, which limits the statistical power to detect publication bias. Therefore, these results should be interpreted with caution, although sensitivity analyses support the overall robustness of the findings.

Note: Red diamonds represent the observed studies reporting hazard ratios for breast cancer mortality in HR-positive patients. The hollow red diamond represents one imputed study added by the Trim and Fill method to account for potential publication bias. The vertical blue dashed line indicates the pooled hazard ratio from the random-effects meta-analysis (HR = 0.765). The Y-axis shows the standard error of log(HR), with studies of higher precision at the top. This plot demonstrates that even after accounting for a potential missing study, the overall protective effect of statin use on mortality remains robust.

#### 3.3.4 Subgroup analysis: impact of statin use on mortality in different stage HR+ breast cancer

**Figure 5:**
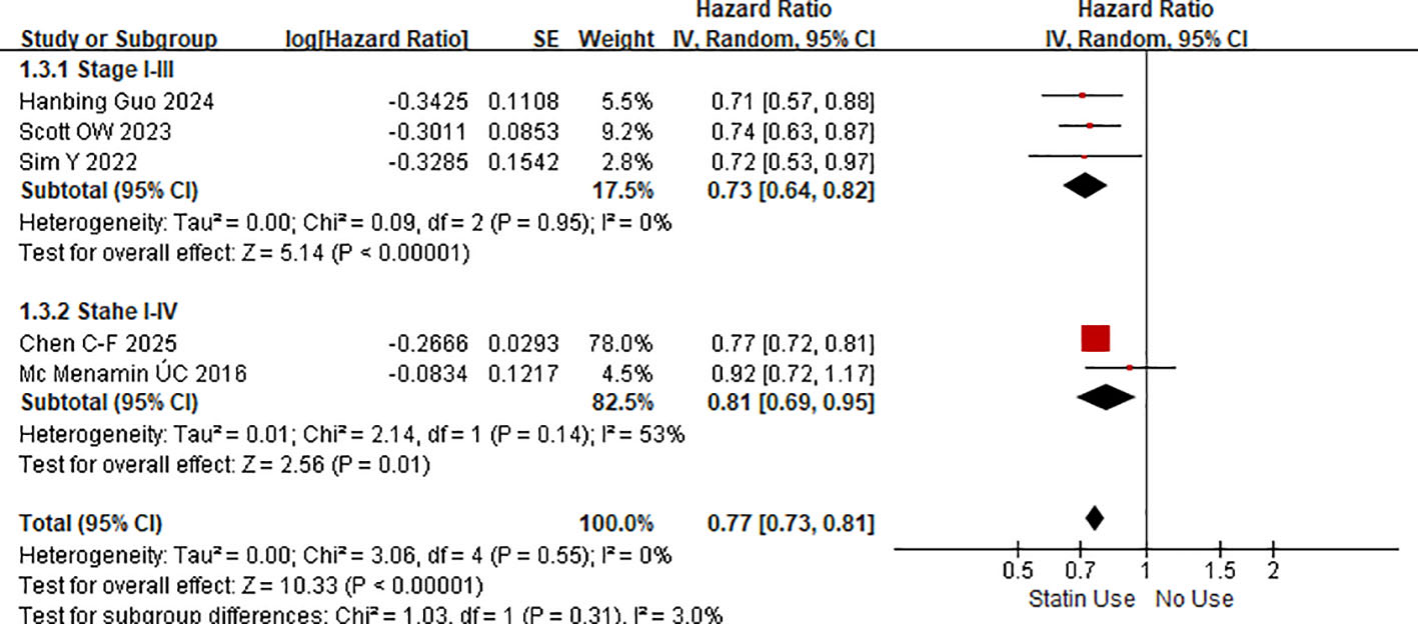
The forest plot compares the impact of statin use on mortality in different stage HR+ breast cancer.

Subgroup analysis was performed according to cancer stage to evaluate the association between statin use and mortality in HR-positive breast cancer patients (**Figure 5**).

For early-stage patients (stage I–III), statin use was associated with a significantly reduced risk of mortality, with a pooled HR of 0.73 (95% CI: 0.64–0.82). No heterogeneity was observed among the included studies (I² = 0%, P = 0.95), indicating high consistency of the results.

For studies including stage I–IV patients, statin therapy was also associated with a significantly lower mortality risk (HR = 0.81, 95% CI: 0.69–0.95, P = 0.01), although moderate heterogeneity was present (I² = 53%).

Overall, the pooled analysis demonstrated that statin use was associated with a 23% reduction in mortality risk among HR-positive breast cancer patients (HR = 0.77, 95% CI: 0.73–0.81, P < 0.00001), with no significant heterogeneity across studies (I² = 0%).

#### 3.3.5 BCSS subgroup analysis

A subgroup analysis was conducted to evaluate the association between statin use and BCSS. As shown in the forest plot, statin therapy was associated with a significantly improved BCSS compared with non-use.

The pooled HR was 0.76 (95% CI: 0.68–0.85, P < 0.00001), indicating a 24% reduction in breast cancer–specific mortality risk among patients receiving statin therapy.

**Figure 6:**
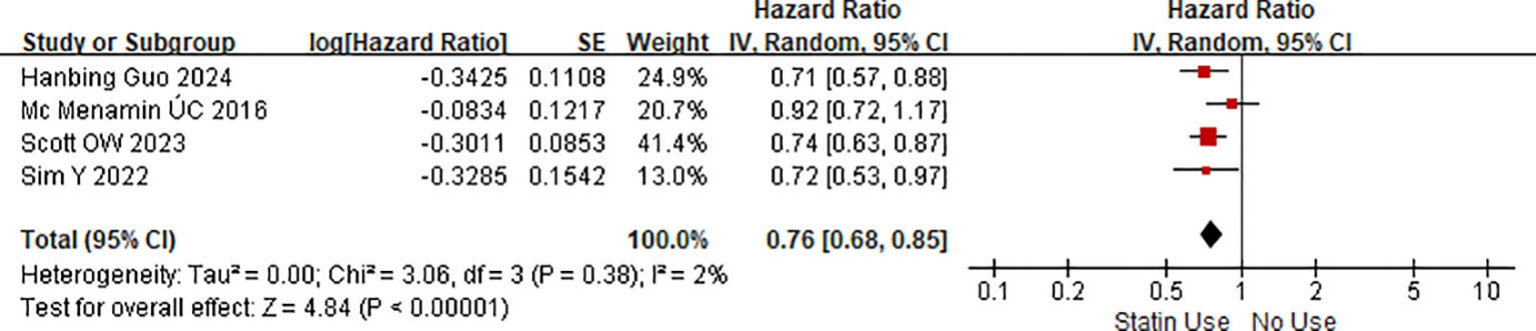
Forest plot of statin use versus no statin use on breast cancer–specific survival using a random-effects model.

Heterogeneity among the included studies was minimal (Tau² = 0.00; Chi² = 3.06; df = 3; P = 0.38; I² = 2%), suggesting high consistency of results across studies (**Figure 6**).

Overall, these findings indicate that statin use may be associated with improved breast cancer–specific survival.

#### 3.3.6 Sensitivity analysis

**Figure 7:**
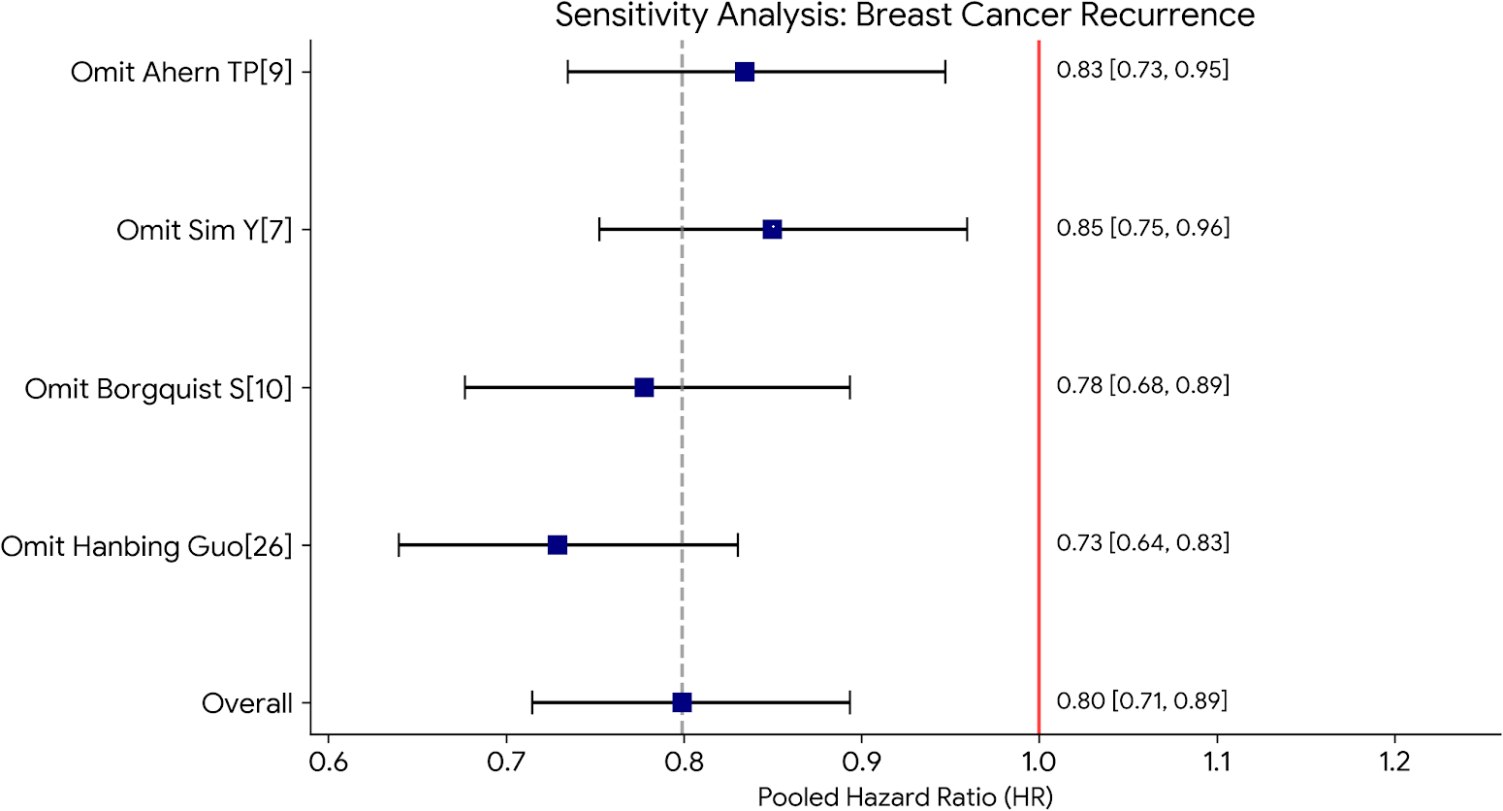
Leave-one-out sensitivity analysis for the association between statin use and breast cancer recurrence in HR-positive breast cancer.

**Figure 8:**
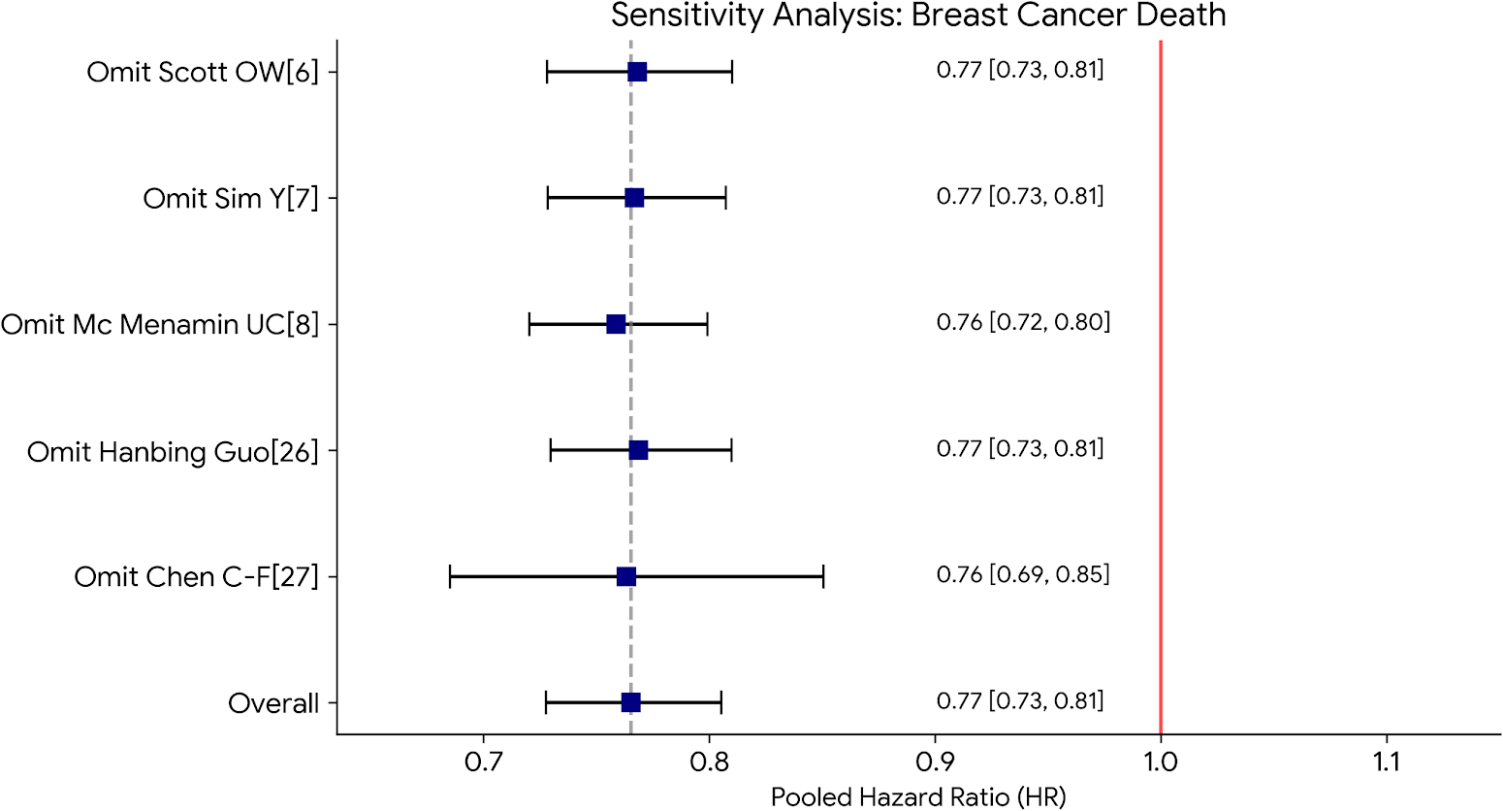
Leave-one-out sensitivity analysis for the association between statin use and breast cancer mortality in HR-positive breast cancer.

Leave-one-out sensitivity analyses were performed to evaluate the robustness of the meta-analysis results (**Figures 7**, **8**). In this approach, each study was sequentially removed and the pooled HR was recalculated.

For breast cancer recurrence (**Figure 7**), sequential omission of individual studies resulted in pooled HRs ranging from 0.73 to 0.85, all of which remained statistically significant and below the null value (HR = 1.0). The overall pooled estimate was HR = 0.80 (95% CI: 0.71–0.89). These results indicate that the association between statin use and reduced recurrence risk was stable and not driven by any single study.

Note: Sensitivity analyses using leave-one-out method confirm that the overall recurrence HR is robust to exclusion of any single study.

For breast cancer mortality (**Figure 8**), the pooled HR remained highly consistent when each study was excluded in turn, with estimates ranging from 0.76 to 0.77. The overall pooled effect was HR = 0.77 (95% CI: 0.73–0.81). No individual study substantially altered the pooled estimate, confirming the robustness of the mortality findings.

Overall, the sensitivity analyses demonstrated that the meta-analysis results were stable, and no single study disproportionately influenced the conclusions regarding the association between statin use and improved outcomes in HR-positive breast cancer.

## 4 Discussion

Current studies suggest statins may significantly improve prognosis in breast cancer patients, particularly in early-stage HR+ cases. A study published in the journal *Cancer* (11) pointed out that the overall survival rate of breast cancer patients taking statins could be relatively increased by 30%, while the BCSS rate could be relatively increased by 58%. Furthermore, as a reflection of general prognostic trends, research on the recurrence rate of breast cancer demonstrated that statin users displayed longer mean RFS (16.6 vs 10.2 years, P = 0.028); after data had been adjusted for patient and disease characteristics, statin users maintained a lower risk of recurrence (6, 12, 13). Furthermore, this RFS benefit appeared to be confined to the use of lipophilic statins (HR 0.72; 95% CI 0.59–0.89), as hydrophilic statin use was not associated with improvement in recurrence-free survival (HR 0.80; 95% CI 0.44–1.46) (14).

In this updated meta-analysis encompassing seven studies and 267,913 patients, statin use was associated with a 23% reduction in recurrence risk (HR = 0.77, 95% CI: 0.61–0.98, P = 0.03). Substantial heterogeneity was observed for recurrence (I² = 76%), reflecting differences in study populations, follow-up durations, and outcome ascertainment. Although a meta-regression by statin solubility could, in principle, help explore sources of heterogeneity, it was not feasible in this analysis for two reasons: (1) only one study (Ahern TP 2011) reported recurrence outcomes stratified by lipophilic versus hydrophilic statins, and (2) the total number of studies included in the recurrence analysis was limited (n = 4), which is insufficient for stable and reliable meta-regression. Other recurrence studies, including Sim 2022 and Hanbing Guo 2024, reported only overall recurrence HRs without solubility-specific data. To address heterogeneity, we conducted leave-one-out sensitivity analyses (**Figures 7, 8**), which confirmed that the pooled HR remained robust regardless of study exclusion. Qualitative evaluation suggests that the protective effect on recurrence is likely driven by lipophilic statins, whereas hydrophilic statins appear less effective. Therefore, the pooled recurrence HR should be interpreted cautiously, as it may mask differences between statin types, and future studies providing recurrence outcomes stratified by statin solubility are warranted. Conversely, for mortality outcomes, the combined effect for stage I–IV HR+ breast cancer was highly significant, demonstrating a 23% reduction in overall mortality risk (HR = 0.77, 95% CI: 0.73–0.81, P < 0.00001). Notably, this survival benefit exhibited minimal heterogeneity (I² = 0%), highlighting a highly robust and consistent protective effect across diverse patient cohorts.

### Possible Sources of Heterogeneity

The substantial heterogeneity observed in the recurrence analysis likely stems from differences in the study populations among the included cohorts: Disease Stage: Mc Menamin ÚC (7) and Chen C-F (10) included patients with stages I–IV invasive breast cancer, while the other five studies focused solely on stages I–III HR+ breast cancer. Differences in disease stage likely contributed to heterogeneity across the included studies. Importantly, in the present meta-analysis, studies including stage IV patients were retained only for survival-related analyses and were not included in the recurrence analysis. The recurrence analysis was restricted to stage I–III or otherwise non-metastatic HR+ populations. Nevertheless, variation in stage composition across studies may still have influenced the survival estimates. In addition, incomplete reporting of baseline T stage, nodal status, and metastatic burden in some studies may have introduced residual between-study heterogeneity. Age Distribution: Age demographics varied considerably. In Ahern TP (8), statin users were mostly aged 50–69. Mc Menamin ÚC (7) reported statin users primarily aged 60–79, whereas non-users were predominantly aged 50–69. Sim Y (6) had a younger non-user group compared to statin users. In Borgquist S (9), the statin user group represented 6% of those under 65 years old and 11% of those 65 and older. Overall, statin users tended to be older, which is a common trend in clinical practice. Research Methods: The 7 studies include populations of different ethnicities and regions (e.g., US, UK, Sweden, Denmark, Taiwan), and the confounding factors adjusted for are not perfectly consistent. Ahern TP (8), utilizing a prospective cohort design, can better establish causal relationships compared to the retrospective studies, reducing selection and recall biases. Specific Differences in Outcome Evaluation: Ahern TP (8) found that lipophilic statins significantly reduced recurrence, whereas hydrophilic statins showed no significant effect. Borgquist S (9) reported improved outcomes primarily in patients treated with letrozole, without differentiating lipid-lowering drug types. In contrast, Sim Y (6) conducted no subgroup analyses regarding drug types or endocrine therapy. These methodological inconsistencies potentially increase heterogeneity.

Publication bias was evaluated for both recurrence and mortality outcomes using funnel plots and Egger’s regression tests. For recurrence, the funnel plot (**Figure 3**) showed an approximately symmetric distribution of studies (circles) around the pooled effect estimate, and Egger’s test indicated no significant small-study effects (P = 0.39), suggesting minimal publication bias. For mortality outcomes, the funnel plot of observed studies (**Figure 4**) did not show obvious asymmetry; however, Egger’s test was statistically significant (P = 0.009), indicating potential small-study effects. To address this, a Trim and Fill analysis was conducted, which imputed one hypothetical study. The adjusted pooled hazard ratio changed minimally to HR = 0.765 (95% CI: 0.728–0.805), demonstrating that the overall protective effect of statin use on mortality remained robust even after accounting for potential publication bias. It is important to note that the number of studies included in both recurrence and mortality analyses was fewer than ten, limiting the statistical power to detect publication bias. Nonetheless, the sensitivity analyses and Trim and Fill adjustment support the robustness of the observed effects, particularly for mortality, while recurrence results appear largely unbiased. Mechanisms by Which Statins Improve Prognosis in Breast Cancer.

HR+ breast cancer patients taking endocrine therapy may experience elevated blood lipid levels due to the reduction of estrogen levels. Statins, which lower blood lipid levels, could potentially mitigate the postoperative incidence of cardiovascular and cerebrovascular diseases in HR+ patients. On a molecular level, statins primarily reduce cholesterol levels by inhibiting HMG-CoA reductase (14–16). This action helps to diminish the lipid-rich environment required by breast cancer cells, thereby depriving tumors of essential metabolic precursors and inhibiting tumor growth and spread. Specifically, statins have demonstrated the capacity to inhibit the proliferation and migration of breast cancer cells, thereby diminishing their invasive potential (11, 17, 18). This effect is likely attributable to statins’ multifaceted mechanisms, which include the regulation of the cell cycle, the induction of apoptosis, and the suppression of tumor angiogenesis. Furthermore, statins may confer a beneficial impact on prognosis by bolstering the immune system’s capacity to counteract tumor growth (19). Specifically, statins have been shown to facilitate the maturation and activation of dendritic cells, which are pivotal for the immune system’s recognition and subsequent elimination of tumor cells (20–23).

Limitations of This Study: Subgroup Analysis Constraints: The meta-analysis data were not sufficiently detailed to conduct a thorough subgroup analysis based on specific endocrine therapy medications or lipid-lowering drugs (lipophilic vs. hydrophilic). Additionally, it did not allow for classification based on tumor size, axillary lymph node metastasis, or other immunohistochemical results. Study Design: The meta-analysis largely relied on retrospective data, which is more susceptible to selection and recall biases compared to prospective trials. Lack of Long-Term Data: Some included studies lacked extended long-term follow-up data, which is essential for fully understanding the prolonged effects of statin use on survival. Dosage and Duration: Information regarding the cumulative dosage and precise duration of statin use was not uniformly detailed across the studies, which are critical factors that could influence breast cancer outcomes (24).

Conclusion and Future Directions.

In conclusion, this study suggests that statin use is associated with improved survival outcomes in HR+ breast cancer and may also be associated with reduced recurrence risk. The survival findings were highly consistent across studies, whereas the recurrence findings were more heterogeneous and should be interpreted cautiously. These findings support further prospective investigation rather than definitive clinical adoption, and larger multicenter studies are needed to clarify the magnitude of benefit, optimal statin subtype, treatment duration, and the patient subgroups most likely to benefit.

## Data Availability

The original contributions presented in the study are included in the article/supplementary material. Further inquiries can be directed to the corresponding author.
